# Effect of Supplementation of Chlorogenic Acid to High-Fat Diet on Growth, Lipid Metabolism, Intestinal and Hepatic Histology, and Gut Microbiota of Spotted Sea Bass (*Lateolabrax maculatus*)

**DOI:** 10.3390/metabo13101067

**Published:** 2023-10-10

**Authors:** Jianrong Ma, Lumin Kong, Sishun Zhou, Hao Lin, Yi Lin, Huihui Qin, Zhongying Long, Longhui Liu, Zhangfan Huang, Zhongbao Li

**Affiliations:** 1 Fisheries College, Jimei University, Xiamen 361021, China; 202112951045@jmu.edu.cn (J.M.);; 2 Fujian Provincial Key Laboratory of Marine Fishery Resources and Eco-Environment, Xiamen 361021, China

**Keywords:** chlorogenic acid, high-fat diet, lipid metabolism, histology, spotted sea bass

## Abstract

The study investigated the impact of chlorogenic acid (CGA) supplementation in a high-fat diet (HFD) on growth, lipid metabolism, intestinal and hepatic histology, as well as gut microbiota in spotted sea bass. A total of 540 fish were fed six experimental diets, including a normal fat diet (NFD), a high-fat diet (HFD), and HFD supplemented with 100, 200, 300, and 400 mg/kg CGA (named HFD1, HFD2, HFD3, and HFD4, respectively) for 7 weeks. The results showed that HFD feeding increased growth and hepatic lipid deposition compared to that in the NFD group. Inclusion of 300 mg/kg CGA in HFD decreased the HFD-induced hyperlipemia (*p* < 0.05). Additionally, compared to the HFD group, the HFD4 group showed significant reductions in serum aspartate transaminase (AST) and alanine transaminase (ALT) levels as well as hepatic malondialdehyde (MDA) content, while also improving liver total antioxidant capacity (T-AOC) (*p* < 0.05). In the CGA-containing groups, hepatocytes were arranged more neatly than those in the HFD group, and there was a reduction in lipid deposition and hemolysis in the liver. Supplementation of CGA had effects on intestinal structure including an increase in mucosal thickness, as well as villus number and width. The diversity of intestinal flora in the CGA-containing groups was higher than those in the HFD group, and supplementation of 200 mg/kg CGA significantly increased the abundance of intestinal bacteria (*p* < 0.05). HFD4 feeding increased the intestinal Bacteroidetes to Firmicutes ratio and decreased the abundance of *Vibrio*. The highest value abundance of Actinobacteriota was found in the HFD2 group. Overall, HFD caused negative effects, and supplementation of 200–400 mg/kg CGA to HFD improved fat deposition, lipid metabolic disorders and liver and gut histology, and increased gut bacterial diversity in spotted sea bass.

## 1. Introduction

In intensive aquaculture, the use of high-fat diets (HFDs) is the current trend due to their protein-sparing and growth-promoting impacts [1,2,3]. Lipids not only provide energy and essential fatty acids, but also maintain nutrient solubility, absorption, and transport in fish [4]. In fact, studies have shown that increasing dietary lipids can improve fish growth rates within the fat requirement [5,6]. However, the high proportion of dietary fat levels has resulted in a series of adverse effects on fish. For instance, a HFD will result in hepatic fat accumulation, accompanied by fatty liver and lipid metabolism disorders that may impair growth performance, health status and nutritional quality of fish, and even cause economic losses [7,8]. It is reported that spotted sea bass fed a HFD resulted in a significant increase in the content of triglycerides (TG) and total cholesterol (TC) in the serum [9]. In addition, research on tilapia indicated that a HFD led to destruction of the integrity of the hepatocyte structure, significant inflammatory infiltration, and steatosis in liver cells [10]. It has been reported that a HFD injures gut structures and disrupts intestinal flora homeostasis in juvenile Nile tilapia [11,12], and the villi in the foregut of *Cyprinus carpio* was significantly reduced after consuming a HFD [13]. Additionally, a HFD has been demonstrated to damage the intestinal health in juvenile rice field eel (*Monopterus albus*) [14]. Therefore, it is necessary to find effective solutions to mitigate the negative effects induced by HFD in aquaculture.

Chlorogenic acid (CGA) is a ubiquitous phenolic acid abundant in *Eucommia ulmoides*, honeysuckle, and other plants. It has beneficial effects on promoting growth performance, improving antioxidant capacity [15], alleviating colon mucosal damage, improving intestinal morphology and structure integrity [16], as well as reducing the infiltration of inflammatory cells and lipid deposition in rat liver caused by HFD [17]. Furthermore, many studies have reported its function in regulating lipid metabolism. For example, previous research reported that CGA can inhibit the growth of adipocyte population [18]. In addition, CGA regulated lipid metabolism through suppressing liver fat synthesis, promoting fatty acid oxidation, stimulating AMPK activation, and regulating fatty acid composition [19]. In *Micropterus salmoides*, dietary CGA can enhance the expression of lipolysis and lipid transport genes, reduce plasma lipid levels, and thus affect the hepatic lipid metabolism [20]. Supplementing a HFD with CGA can reduce serum levels of T-CHO and LDL-C, while increasing HDL-C levels in grass carp [21]. Moreover, in vitro, CGA significantly increased the abundance of Firmicutes and Bacteroides [22]. Dietary CGA can regulate gut microbiota in growing pigs [23]. And, CGA increased the relative abundance of gut microbiota, Fusobacteria and Firmicutes, as well as reduced the relative abundance of Proteobacteria [24].

Spotted sea bass, *Lateolabrax maculatus*, is a valuable species for cultivation in coastal and estuarine areas of China, Japan, and the Korean Peninsula due to its rapid growth, high nutritional value, delicious meat, wide salinity and temperature ranges [25,26,27]. Meanwhile, it is sensitive to HFD, which can easily lead to fatty liver and other diseases that threaten the healthy development of spotted sea bass aquaculture [9]. Considering the functional properties of CGA, the effects of dietary CGA on spotted sea bass fed a HFD have not been well elucidated and require further exploration. Therefore, the aim of the current study was to investigate the effect of CGA supplementation on growth, lipid metabolism, intestinal and hepatic histology, as well as gut microbiota in spotted sea bass fed a HFD. This study highlights the potential of CGA to mitigate the negative effects resulting from the use of HFD in aquaculture.

## 2. Materials and Methods

### 2.1. Animal Ethics

This trial was supported by the Animal Ethics Committee of Jimei University. The authors have followed all international, national, and/or institutional guidelines applicable to the care and use of animals.

### 2.2. Experimental Diets

Six diets were prepared for the experiment. The normal fat content diet (NFD), which contained approximately 11.9% lipid level, and the high-fat diet (HFD), which contained around 17% lipid level, were considered as control groups. The other four diets were HFD1, HFD2, HFD3, and HFD4, which were supplemented with 100, 200, 300, and 400 mg/kg of CGA in the HFD, respectively. The CGA provided by Changsha Shanghe Biotechnology Co., Ltd. (Changsha, China) has an effective content of ≥98%. The addition level of CGA in this experiment was referred from previous research reports on grass carp and *Cyprinuscarpio* var. Jian [21,28]. The processing procedures are as follows: all diet ingredients were thoroughly mixed, pressed into particles with a diameter of 2.5 mm using a pellet mill, dried in an oven at 55 °C, and then stored at −20 °C until they were used. Table 1 provides the dietary formulation and nutrient composition of the diets used in this study.

### 2.3. Experimental Fish and Feeding Trial

Spotted sea bass were obtained from a commercial hatchery in Zhangzhou (Fujian, China) and acclimated to experimental conditions for 2 weeks in 1200 L tanks. The experiment was conducted in a recirculating aquaculture system at Jimei University Fishery Experimental Station (Xiamen, China). After domestication, 540 fish of similar size (5.42 ± 0.10 g) were randomly assigned to 18 experimental tanks (200 L) with 30 fish per tank. Each diet was divided into 3 experimental tanks, and the fish were fed twice a day (at 8:30 and 17:30) until they appeared satiated. During the experimental period, the dissolved oxygen remained at around 7 mg/L, the temperature was sustained at approximately 28 ± 1.5 °C, pH was kept between 7.5–8.5 units, and salinity ranged from 0.5 to 2.0.

### 2.4. Sample Collection

After the feeding test, the fish were food deprived for 24 h and then anesthetized with eugenol (1:10,000). The total number and weight of fish in each tank were recorded to calculate the weight gain rate (WG) and feed conversion rate (FCR). Subsequently, eleven fish were selected from each tank and their weight and body length were recorded to calculate the condition factor (CF) and specific growth rate (SGR). Then, a 1 mL syringe was used to collect blood from the tail veins of the aforementioned eleven fish, and the serums were obtained by centrifugation at 4 °C (3000× *g* rpm for 10 min). The serums were aliquoted and stored at −80 °C for later detection of biochemical indexes. The eleven fish livers were collected and weighed in order to calculate the hepatosomatic index (HSI). Subsequently, nine liver tissues were averagely divided into three tubes and stored in a −80 °C refrigerator for analysis of antioxidant capacity, and two liver tissues were fixed in a 4% paraformaldehyde solution for the preparation of Oil Red O sections and hematoxylin and eosin (H&E) stained sections. Finally, three foreguts, randomly collected from the same eleven fish, were distributed into three tubes for storage at −80 for gut microbiota analysis. Moreover, two additional foregut tissues were fixed in a 4% paraformaldehyde solution for preparation of H&E stained sections.

### 2.5. Growth Performance Parameters

Weight gain (WG), specific growth rate (SGR), feed conversion rate (FCR), hepatosomatic index (HSI), and condition factor (CF) were determined using the following equations: Weight gain (WG, %) = (final mean body weight − initial mean body weight)/initial mean body weight × 100; Specific growth rate (SGR, %/d) = ((ln (final mean body weight) − ln (initial mean body weight))/49 × 100; Feed conversion rate (FCR) = total feed intake/(final mean body weight − initial mean body weight); Hepatosomatic index (HSI, %) = liver wet weight/fish body wet weight × 100; CF (g/cm^3^) = fish body wet weight/(individual fish body length)^3^ × 100.

### 2.6. Plasma Biochemical Indexes and Hepatic Antioxidant Capacity Analysis

The contents of total cholesterol (TC), triglycerides (TG), high-density lipoprotein (HDL-C), low-density lipoprotein (LDL-C), aspartate aminotransferase (AST), and alanine aminotransferase (ALT) in serum were detected using a microplate reader (BioTek, Winooski, VT, USA). Approximately 0.1 g of liver tissue was weighed and transferred into 1.5 mL tubes, followed by the addition of nine times the volume of ice-cold saline (8.6 g/L NaCl in dd H_2_O) and magnetic beads for homogenization. The homogenate was subjected to centrifugation at 4 °C (2500× *g* rpm for 10 min), and the resulting supernatants were aliquoted into tubes and stored at −80 °C until further analysis. Total antioxidant capacity (T-AOC) was assayed using the microplate reader. Malondialdehyde (MDA) was measured using a spectrophotometer (UV-1200, Shanghai, China). The analysis of indexes was conducted in accordance with the protocols provided by the commercial kits (Nanjing Jiancheng Bioengineering Institute, Nanjing, China).

### 2.7. Oil Red O Staining for Liver

Liver samples were fixed in a 4% paraformaldehyde solution for 24 h, dehydrated with 30% sucrose, and then frozen sectioned. After being rewarmed and dried, the frozen sections were fixed in the 4% paraformaldehyde solution for 15 min. Subsequently, the sections were briefly washed in sterile water, dipped in 75% alcohol, and then stained with the Oil Red O solution for 8–10 min. Afterwards, hematoxylin was applied for staining purposes. All sections were observed under a microscope (Nikon, Tokyo, Japan).

### 2.8. Liver and Gut Histology Analysis

Liver and foregut samples were fixed in a paraformaldehyde fixative for 24 h. The samples underwent dehydration, paraffin embedding, sectioning into slices (4–6 µm), and subsequent staining with hematoxylin and eosin (H&E). Images of the liver and foregut sections were obtained using a light microscope (Nikon, Tokyo, Japan). The villus length (VL), villus width (VW), and muscular thickness (MT) of the foregut were measured using ImageJ (NIH) software.

### 2.9. Analysis of Gut Microbiota

Gut microbiota was analyzed using 16S rDNA gene sequencing. The DNA was extracted from the foregut contents of three fish in each diet using the TGuide S96 magnetic fecal DNA extraction kit (TianGen, Beijing, China), following the manufacturer’s instructions. Subsequently, the V3–V4 region of 16S rDNA was amplified via PCR using the primers 338F (ACTCCTACGGGAGGCAGCAG) and 806R (GGACTACHVGGGTWTCTAAT), and then sequenced on the Illumina NovaSeq 6000 platform. Trimmomatic (v 0.33) and Cutadapt (v 1.9.1) were used for quality control procedures on raw pair-end reads and for identification and removal of primer sequences, respectively. USEARCH (v 10.0) was utilized for splicing double-ended reads, eliminating chimeras, and then assembling an operational taxonomic unit (OTU). The qualified reads underwent clustering analysis using USEARCH (v 10.0) with a similarity threshold of 97%, and OTUs filtering was applied with a sequence number threshold of 0.005%. QIIME2 software was used to assign the representative sequences of each OTU to a classification level in the Silva.138 database. ACE and Chao were used to represent bacterial abundance and Simpson and Shannon were utilized to assess bacterial diversity.

### 2.10. Statistical Analysis

ANOVA was performed to test the differences among treatments at a *p*-value of <0.05, followed by Duncan’s multivariate analysis for comparison between multiple groups using SPSS 25 (Chicago, IL, USA). The data were expressed as means ± SD (*n* = 3). A *p*-value of <0.05 was considered to be statistically significant.

## 3. Results

### 3.1. Growth Performance, Feed Intake, and Morphometric Parameters

Table 2 shows the results of WG, SGR, HSI, FCR, and CF. No significant differences were found in WG, SGR, and HSI among the six groups (*p* > 0.05). The fish fed with HFD4 diet exhibited the highest WG and SGR (*p* > 0.05). The FCR was significantly lower in the CGA-containing groups compared to the NFD and HFD groups (*p* < 0.05), and it was significantly lower in the HFD3 group compared to both the HFD2 and HFD4 groups (*p* < 0.05). The CF was significantly higher in the HFD2 and HFD3 groups than in the HFD group (*p* < 0.05).

### 3.2. Lipid Levels in Plasma

**Figure 1:**
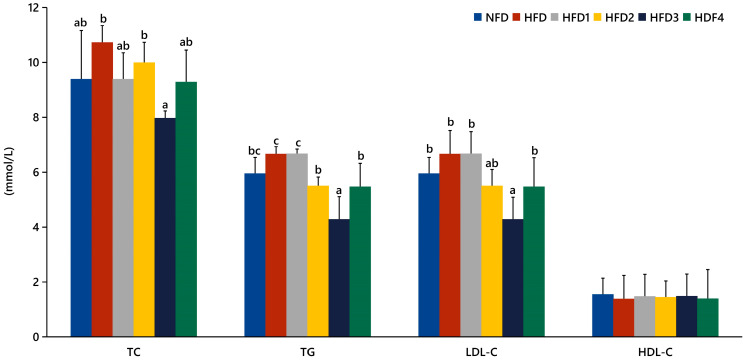
Effects of CGA on serum lipid levels of spotted sea bass. TC: total cholesterol; TG: total triglyceride; LDL-C: low-density lipoprotein; HDL-C: high-density lipoprotein. The same index with different letters indicates significant difference (*p* < 0.05), while with the same letter or no letter means no significant difference (*p* > 0.05).

The serum levels of TC, TG, LDL-C, and HDL-C in spotted sea bass fed experimental diets are shown in Figure 1. The HFD3 group showed significantly lower levels of TG, TC, and LDL-C compared to the HFD group (*p* < 0.05). There were no significant differences in serum HDL-C levels between the groups (*p* > 0.05).

### 3.3. Liver Antioxidant Capacity and Serum Biochemical Indexes

Table 3 shows the results of hepatic antioxidant capacity and serum biochemical indexes. The HFD2 and HFD3 groups had significantly lower MDA content than the HFD group (*p* < 0.05), but their MDA content was not significantly different from that of the NFD group (*p* > 0.05). The T-AOC activity in the HFD4 group was significantly higher than that in the HFD group (*p* < 0.05), but there was no significant difference compared to the NFD group (*p* > 0.05). HFD feeding increased the levels of serum AST and ALT compared to the NFD group. Fish fed HFD3 and HFD4 exhibited significantly lower levels of ALT and AST compared to the HFD group (*p* < 0.05).

### 3.4. Oil Red O Stained Sections of Liver

**Figure 2:**
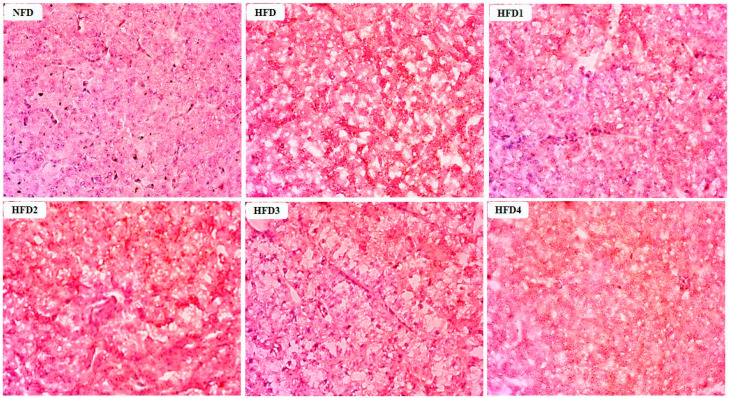
Effects of CGA on the accumulation of lipid droplets in the liver of spotted sea bass by Oil Red O staining analysis. Red dots indicate the lipid.

The results of Oil Red O stained sections of liver are shown in Figure 2. These sections were analyzed according to the methods of previous studies [29]. The livers of the NFD and CGA-containing groups showed a few lipid droplets, while the liver of the HFD group exhibited a substantial accumulation of lipid droplets.

### 3.5. Liver and Gut Histology

**Figure 3:**
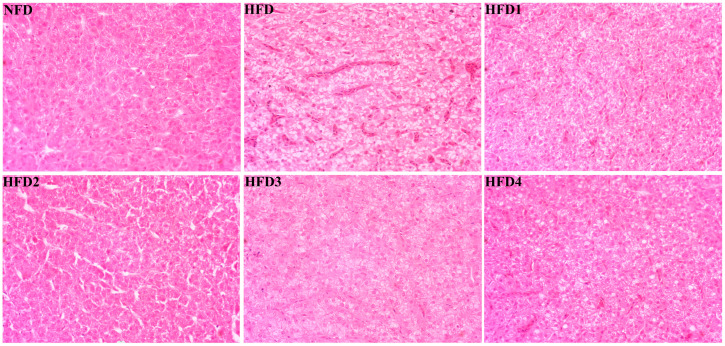
Effects of CGA on liver histology of spotted sea bass. Magnification is 40×.

**Figure 4:**
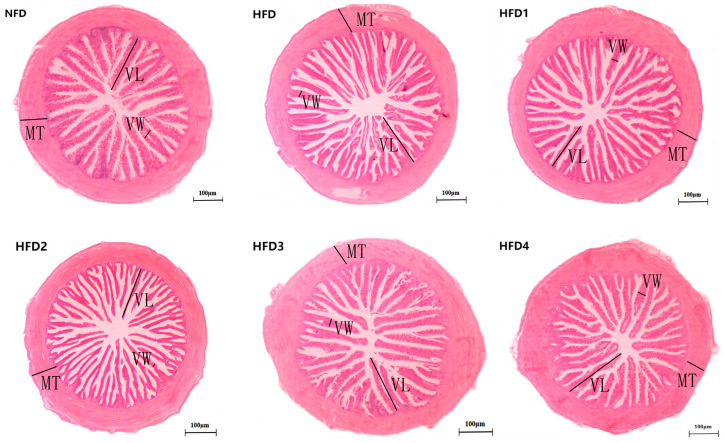
The gut histology of spotted sea bass fed with different diets. MT, VW, and VL represent mucosal thickness, villus width, and villus length respectively. Magnification is 100× and the scale bar is 100 µm.

**Figure 5:**
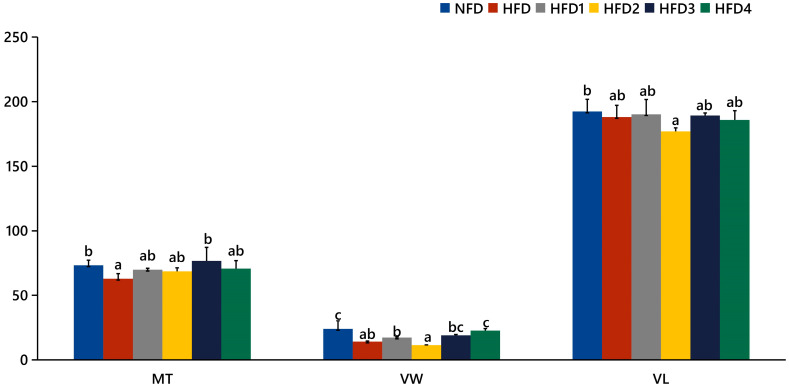
Statistical analysis of intestinal mucosal thickness (MT), villus width (VW), and villus length (VL). The same index with different letters indicates significant difference (*p* < 0.05), while with the same letter or no letter means no significant difference (*p* > 0.05).

As presented in Figure 3, the liver tissues of the NFD group showed normal histology, characterized by regular hepatocytes and clear boundaries between cells. In contrast, the livers of fish fed a HFD displayed several liver lesions, including congestion of sinusoid, lipidosis, and destruction of hepatocyte structure. Meanwhile, dietary CGA improves these pathological changes to maintain the normal liver structure. Morphological parameters and histological sections of the intestine are presented in Figure 4 and Figure 5. Fish fed HFD3 (76.75 ± 10.40) showed a significant increase in mucosal thickness compared to those fed a HFD (62.85 ± 3.95) (*p* < 0.05). The villus width of fish fed HFD4 (22.68 ± 1.54) was significantly higher than those fed a HFD (14.22 ± 0.40) (*p* < 0.05). There were no significant differences in villus length among all groups, while the number of intestinal villi increased after the CGA treatment. Moreover, the addition of CGA to a HFD can improve the damage of intestinal villi.

### 3.6. Gut Microbiota Analysis

**Figure 6:**
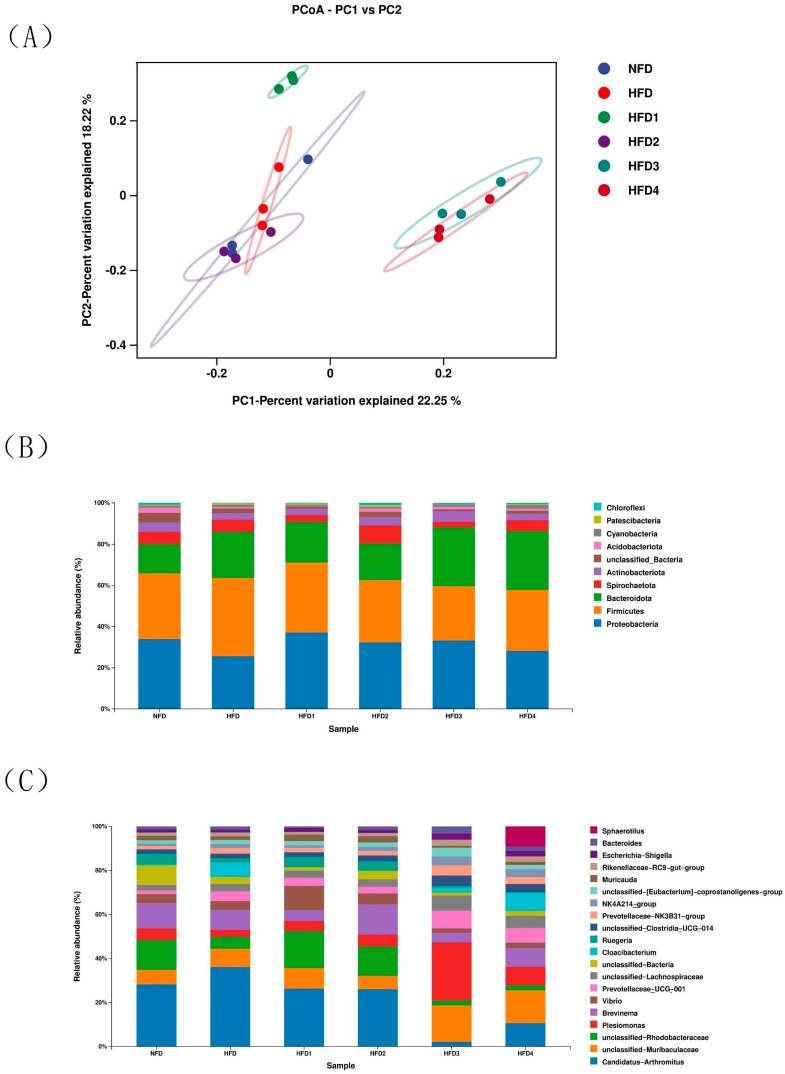
The intestinal microbiota of spotted sea bass fed with different diets. (**A**) Principal coordinates analysis (PCoA), relative abundance at phylum (**B**), and genus (**C**) level of the gut microbiota.

The results of ACE, Chao, Simpson and Shannon are shown in Table 4. The Chao and ACE were significantly higher in the HFD2 group than in the HFD group (*p* < 0.05), reaching their highest values. The Simpson and Shannon of the CGA-containing groups exceeded those of the HFD group (*p* > 0.05). Moreover, the overall structure of the gut microbiota in CGA-containing groups significantly differed from that of the HFD group, as shown by principal coordinate analysis (PCoA), indicating the effects of CGA supplementation on intestinal microflora (Figure 6A).

The composition of gut microbiota of sea bass is shown in Figure 6B,C and Table 5. At the phylum level (Figure 6B), Proteobacteria, Bacteroidetes, Firmicutes, and Actinobacteria dominated the species distribution of the gut microbiota. In comparison to the NFD group, the HFD group exhibited a decreased relative abundance of Proteobacteria and Actinobacteriota, while showing an increased relative abundance of Firmicutes and Bacteroidota. Moreover, the ratio of Firmicutes in HFD1 and HFD2 groups was close to that in the NFD group. The HFD1 group showed a higher ratio of Proteobacteria than the HFD group (*p* < 0.05). In addition, Bacteroidota abundance increased in HFD3 and HFD4 groups (*p* < 0.05), but decreased in HFD1 and HFD2 groups compared to that in the HFD group. The highest value of Actinobacteriota was observed in the HFD2 group. At genus level (Figure 6C), the relative abundance of *Vibrio* and *Brevinema* decreased in the HFD3 and HFD4 groups compared to that in the HFD group (*p* > 0.05).

## 4. Discussion

A previous study has confirmed that adding the appropriate amount of CGA to the diet can promote growth performance in *Ctenopharyngodon idellus*, *Cyprinus carpio* var. Jian, and *Trionyx sinensis* [21,28,30]. No significant effect of CGA supplementation on HSI was observed compared to the HFD group, indicating that CGA may have little influence on liver development. Similar results have been observed in a study of grass carp [31]. Furthermore, we observed significant differences in FCR and CF with CGA supplementation, particularly at the 300 mg/kg dose of CGA. The findings were inconsistent with the study of largemouth bass [20]. The varying outcomes may be attributed to the diverse origins of lipids, fermentation durations, and piscine species. These results indicated that CGA plays a positive role in promoting growth in fish.

High serum lipid levels can lead to lipid metabolism disorders and fat accumulation in the liver [32]; therefore, changes in blood lipids are often used as an indicator of the status of lipid metabolism in both the liver and body [33]. In this experiment, the levels of TG, TC, and LDL-C were found to be higher in the HFD group, indicating that HFD caused lipid metabolism disorders to some extent in spotted sea bass. Moreover, fish fed HFD3 showed significant reductions in the levels of TG, TC, and LDL-C compared to those fed the HFD. Similarly, previous studies have shown that CGA can reduce the levels of TC and LDL-C in the serum of grass carp [21]. Additionally, we observed reductions in hepatic fat accumulation in the HFD1 and HFD3 groups compared to the HFD group. In brief, these results demonstrated that supplementation of 300 mg/kg CGA can positively modify lipid metabolism and fat accumulation of spotted sea bass.

In aquatic animals, the liver and intestine are important organs for digestion and metabolism, and their health statuses are essential for the body to perform normal physiological functions [34,35]. Previous research has indicated that HFD can lead to liver and intestinal damage, as well as an imbalance of intestinal flora [10,12,36,37]. Meanwhile, CGA supplementation has shown excellent results in enhancing oxidative capacity, ameliorating intestinal damage, and regulating gut microbiota [38,39,40]. MDA is considered a biomarker that reflects the levels of oxidative stress, indicating not only the extent of lipid peroxidation, but also indirectly the degree of cellular damage [41]. In this study, the HFD group exhibited a higher content of MDA compared to the NFD group, suggesting that consumption of a HFD resulted in hepatic damage. Meanwhile, we observed that the inclusion of 200–300 mg/kg CGA in HFD resulted in a significant reduction in MDA levels compared to the HFD group. A similar outcome has been reported in *Micropterus salmoides* that were fed diets supplemented with CGA [20]. T-AOC is a significant indicator of both enzymatic and non-enzymatic antioxidant activities, with higher values indicating an increased capacity for antioxidants [42]. In our study, HFD4 feeding significantly increased T-AOC activity compared to HFD feeding. Consistently, *Cyprinus carpio* var. Jian fed diets supplemented with CGA showed an increase in T-AOC activity [28]. Moreover, ALT and AST are commonly found in hepatocytes, and their serum levels increase due to damage to hepatocytes [43]. In the current study, the activities of ALT and AST were significantly decreased after adding 400 mg/kg CGA to HFD. Analogously, it was demonstrated that CGA significantly attenuated serum ALT and AST activities in rats [44]. Moreover, histological analysis of the liver showed steatosis, hepatocyte destruction, and disorder in the HFD group. In the CGA-containing groups, hepatocytes exhibited clearer cell profiles and fewer lipid vacuoles. The results indicated that CGA has a highly beneficial effect on liver injury caused by HFD, which were consistent with the previous studies [17]. In brief, these results manifested that dietary supplementation of 200–400 mg/kg CGA can increase antioxidant capacity and reduce damage, thereby improving liver health status.

It is generally believed that when the intestinal lesions, such as epithelial cell shedding, villi shedding, and muscle layer thinning occur, the digestion and absorption capacity of animals will be significantly reduced [45]. The present study has demonstrated that CGA can enhance the density of villi. And, mucosal thickness and villus width were optimized in the HFD3 and HFD4 groups, respectively, exhibiting a significant increase compared to those observed in the HFD group. Similarly, rats fed diets supplemented with CGA also showed improvements in intestinal inflammation and damage [16]. The reason may be related to the fact that most of the CGA mainly remains in the intestine after entering the body. These results indicated that CGA supplementation in HFD can mitigate intestinal damage and enhance the body’s digestion and absorption abilities.

ACE and Chao indexes are directly proportional to flora richness, while Shannon and Simpson indexes are directly proportional to flora diversity [46]. HFD feeding resulted in a reduction in gut bacterial diversity compared to NFD in this study. Moreover, we observed that the ACE and Chao indexes in the HFD2 group reached their highest values, which were significantly greater than those in the HFD group. The CGA-containing groups exhibited higher values for the Simpson and Shannon diversity indexes compared to the HFD group. These results demonstrated that CGA promoted the growth of many intestinal flora and increased the diversity of gut microbiota in spotted sea bass.

The impact of HFD on the gut microbiota of fish has garnered significant attention due to the close relationship between gut microbiota homeostasis and intestinal health [47]. Studies have indicated that the major taxa in spotted sea bass are Proteobacteria, Firmicutes, Actinobacteria, and Bacteroidetes [48], which is consistent with the findings of this experiment. Furthermore, previous research has demonstrated a correlation between high-fat diets and obesity with a decreased ratio of Bacteroidetes to Firmicutes [49,50]. In this study, the addition of 200–400 mg/kg CGA to the HFD increased the Bacteroidetes to Firmicutes ratio compared to that in the HFD group. The findings suggested that CGA may mitigate obesity by modulating the Bacteroidetes to Firmicutes ratio. In addition, the above results of this study indicated that CGA plays an important role in reducing lipid content. Therefore, we proposed that the increase in Bacteroidetes to Firmicutes ratio caused by CGA might be one of the mechanisms of its lipid-lowering function.

Actinobacteria have been reported as a promising probiotic for aquaculture [51]. This study showed that the HFD group decreased the abundance of Actinobacteria compared to the NFD group. However, the fish fed with HFD2 showed an increased abundance of Actinobacteriota compared to those fed a HFD, indicating that CGA can improve the reduction in beneficial intestinal bacteria caused by HFD. At the genus level, *Vibrio* have been reported to be one of the most important pathogenic bacteria causing bacterial diseases in marine and freshwater fish [52]. Moreover, a previous investigation has suggested that CGA may perform its effects on the microbiota by altering the microbial structure and reducing the abundance of certain pathogenic bacterial species [16]. Our study demonstrated that adding 300–400 mg/kg CGA to the HFD resulted in a decrease in *Vibrio*, suggesting that one possible mechanism by which CGA protects intestinal health is through reducing harmful bacteria. Meanwhile, this may also be a reason why CGA alleviates the damage caused by HFD to intestinal structure.

## 5. Conclusions

The study demonstrated that HFD improved the growth of spotted sea bass, but had negative effects on gut and liver histology, antioxidant ability, and lipid metabolism. Inclusion of CGA in the HFD can promote growth, improve tissue structure and liver antioxidant capacity, reduce serum lipid content and liver lipid deposition, as well as increase the diversity of intestinal bacteria. Therefore, our study suggests that supplementation with 200–400 mg/kg of CGA not only promotes growth, but also has beneficial effects in alleviating the negative impacts of HFD on fish.

## Figures and Tables

**Table 1:** Formulation and proximate composition of experimental diets.

	NFD	HFD	HFD1	HFD2	HFD3	HFD4
Ingredients (g/kg)						
Fishmeal	490	490	490	490	490	490
Soya bean meal	235	235	235	235	235	235
Wheat flour	150	100	99.9	99.8	99.7	99.6
Yeast meal	30	30	30	30	30	30
Fish oil	30	80	80	80	80	80
Soybean meal	20	20	20	20	20	20
Phospholipid	10	10	10	10	10	10
Mineral mixture ^a^	6	6	6	6	6	6
Vitamin mixture ^b^	8	8	8	8	8	8
Choline	6	6	6	6	6	6
Dicalcium phosphate	12	12	12	12	12	12
Antioxidant	3	3	3	3	3	3
Chlorogenic acid	0	0	0.1	0.2	0.3	0.4
Proximate composition (% of dry matter)						
Crude Protein	46.91	46.47	46.33	45.96	45.69	46.24
Crude Lipid	11.9	17.00	17.00	17.00	17.00	17.00
Crude Ash	9.11	8.79	9.59	9.77	9.14	8.84

^a^ Mineral premix contains: MnSO_4_·4H_2_O 50 mg/kg, MgSO_4_·H_2_O 4000 mg/kg, CoCl_2_ (1%) 100 mg/kg, KI 100 mg/kg, FeSO_4_·H_2_O 260 mg/kg, CuSO_4_·5H_2_O 20 mg/kg, ZnSO_4_·H_2_O 150 mg/kg, Na_2_SeO_3_ (1%) 50 mg/kg. ^b^ Vitamin premix contains: riboflavin 45 mg/kg, thiamine 25 mg/kg, pyridoxine hydrochloride 20 mg/kg, inositol 800 mg/kg, Vitamin B12 0.1 mg/kg, Vitamin K3 10 mg/kg, nicotinic acid 200 mg/kg, pantothenic acid 60 mg/kg, biotin 1.2 mg/kg, folic acid 20 mg/kg, Vitamin D3 5 mg/kg, vitamin A acetate 32 mg/kg, ethoxyquin 150 mg/kg, α -tocopherol 120 mg/kg.

**Table 2:** Effects of CGA on growth performance of spotted sea bass.

Indexes	NFD	HFD	HFD1	HFD2	HFD3	HFD4
WG (%)	629.82 ± 101.63	651.10 ± 123.79	610.12 ± 99.81	704.26 ± 84.95	651.24 ± 89.85	732.57 ± 71.42
SGR (%/d)	4.04 ± 0.28	4.09 ± 0.33	3.99 ± 0.27	4.25 ± 0.22	4.11 ± 0.25	4.32 ± 0.18
FCR	1.35 ± 0.42 ^c^	1.31 ± 0.01 ^c^	1.14 ± 0.06 ^ab^	1.16 ± 0.02 ^b^	1.07 ± 0.07 ^a^	1.17 ± 0.01 ^b^
CF (g/cm^3^)	1.39 ± 0.09 ^a^	1.36 ± 0.05 ^a^	1.37 ± 0.04 ^ab^	1.43 ± 0.03 ^b^	1.43 ± 0.02 ^c^	1.39 ± 0.02 ^abc^
HSI (%)	0.97 ± 0.08	0.88 ± 0.01	0.87 ± 0.06	0.90 ± 0.08	0.95 ± 0.03	0.95 ± 0.04

Values in the same column with different superscript letters are significantly different (*p* < 0.05).

**Table 3:** Effects of CGA on liver antioxidant capacity and serum biochemical indexes.

Indexes	NFD	HFD	HFD1	HFD2	HFD3	HFD4
MDA (nmol/g prot)	0.70 ± 0.12 ^a^	1.03 ± 0.08 ^c^	0.94 ± 0.02 ^bc^	0.70 ± 0.05 ^a^	0.69 ± 0.09 ^a^	0.87 ± 0.08 ^b^
T-AOC (mmol/gprot)	0.014 ± 0.001 ^b^	0.011 ± 0.002 ^a^	0.012 ± 0.001 ^ab^	0.013 ± 0.002 ^ab^	0.012 ± 0.001 ^ab^	0.014 ± 0.002 ^b^
ALT (U/L)	10.19 ± 1.78 ^b^	12.71 ± 0.90 ^c^	10.76 ± 1.73 ^bc^	10.49 ± 0.90 ^b^	7.76 ± 0.44 ^a^	7.57 ± 0.51 ^a^
AST (U/L)	82.79 ± 9.41 ^ab^	93.82 ± 9.09 ^c^	92.95 ± 6.06 ^c^	88.64 ± 3.30 ^ab^	78.61 ± 2.72 ^a^	74.58 ± 9.98 ^a^

The different superscripts in rows denote significant difference (*p* < 0.05).

**Table 4:** Diversity index of spotted sea bass intestinal microbiota fed with different diets.

Parameters	NFD	HFD	HFD1	HFD2	HFD3	HFD4
ACE	926 ± 78 ^bc^	787 ± 20 ^ab^	690 ± 63 ^a^	937 ± 138 ^c^	645 ± 48 ^a^	657 ± 80 ^a^
Chao	876 ± 181 ^bc^	861 ± 114 ^bc^	701 ± 32 ^ab^	941 ± 149 ^c^	617 ± 58 ^a^	641 ± 84 ^a^
Simpson	0.97 ± 0.02	0.93 ± 0.05	0.95 ± 0.03	0.95 ± 0.02	0.96 ± 0.02	0.97 ± 0.01
Shannon	7.21 ± 0.49 ^b^	6.23 ± 0.62 ^a^	6.47 ± 0.47 ^ab^	6.97 ± 0.22 ^ab^	6.80 ± 0.36 ^ab^	6.89 ± 0.33 ^ab^

The same parameters with different letters indicate significant difference (*p* < 0.05), while with the same letter or no letter means no significant difference (*p* > 0.05). ACE and Chao represent bacterial abundance; Simpson and Shannon represent bacterial diversity.

**Table 5:** The relative abundance of main phyla and genera in the intestinal microbiota of spotted sea bass fed with different diets.

	NFD	HFD	HFD1	HFD2	HFD3	HFD4
phylum						
Proteobacteria	33.05 ± 3.78 ^ab^	25.18 ± 3.30 ^a^	36.39 ± 5.91 ^b^	31.27 ± 5.42 ^ab^	32.73 ± 9.53 ^ab^	27.71 ± 2.83 ^ab^
Firmicutes	30.53 ± 7.65	37.44 ± 5.41	33.13 ± 7.32	29.18 ± 7.70	25.94 ± 4.10	28.94 ± 2.12
Bacteroidota	13.68 ± 0.95 ^a^	22.06 ± 4.90 ^b^	19.30 ± 3.05 ^ab^	17.31 ± 1.42 ^ab^	28.34 ± 4.85 ^c^	28.05 ± 0.97 ^c^
Spirochaetota	4.21 ± 0.53 ^c^	3.16 ± 0.68 ^ab^	3.06 ± 0.24 ^a^	3.92 ± 0.23 ^bc^	5.02 ± 0.36 ^d^	3.11 ± 0.44 ^ab^
Actinobacteriota	5.88 ± 2.02 ^ab^	5.80 ± 1.36 ^ab^	3.28 ± 0.91 ^ab^	8.64 ± 6.17 ^b^	2.67 ± 0.10 ^a^	5.20 ± 2.04 ^ab^
genus						
*Candidatus_Arthromitus*	14.19 ± 7.25 ^abc^	23.18 ± 10.16 ^c^	23.18 ± 10.16 ^c^	15.95 ± 7.87 ^bc^	1.30 ± 0.55 ^a^	6.47 ± 2.92 ^ab^
*Plesiomonas*	2.78 ± 1.97 ^a^	2.10 ± 0.89 ^a^	3.07 ± 1.64 ^a^	3.40 ± 1.80 ^a^	15.95 ± 11.33 ^b^	5.15 ± 1.53 ^a^
*Brevinema*	5.88 ± 2.03 ^ab^	5.80 ± 1.36 ^ab^	3.28 ± 0.91 ^ab^	8.64 ± 6.17 ^b^	2.67 ± 1.00 ^a^	5.20 ± 2.04 ^ab^
*Vibrio*	2.05 ± 0.55 ^a^	2.55 ± 0.10 ^a^	7.34 ± 3.33 ^b^	3.03 ± 0.80 ^a^	1.21 ± 0.65 ^a^	1.50 ± 0.56 ^a^
*Prevotellaceae_UCG_001*	0.88 ± 0.35 ^a^	2.85 ± 0.95 ^bc^	2.47 ± 0.73 ^b^	1.86 ± 0.46 ^ab^	4.94 ± 1.23 ^d^	4.02 ± 0.55 ^cd^

The same phylum or genus with different letters indicates significant difference (*p* < 0.05), while with the same letter or no letter means no significant difference (*p* > 0.05).

## Data Availability

All data are included in the manuscript.
